# How much and what type of exercises and training were provided to people with spinal cord injury as part of usual physiotherapy and occupational therapy in the SCI-MT Trial?

**DOI:** 10.1038/s41393-026-01180-7

**Published:** 2026-02-27

**Authors:** Jackie Chu, Joanne V. Glinsky, Hueiming Liu, Sharon Roberts, Christine Rimmer, Federica Tamburella, Claire Lincoln, Fernanda Di Natal, Lydia W. Chen, Donna Rainey, Vivien Jørgensen, Jessica van der Lede, Charlotte C. M. van Laake-Geelen, Mark McDonald, Emilie J. Gollan, Sue Paddison, Chris Bell, Kristine Oostra, Lot Van Roey, Marsha Ben, Keira T. Tranter, Giorgio Scivoletto, Annemie I. Spooren, Janneke K. Stolwijk, Lisa A. Harvey

**Affiliations:** 1 Kolling Institute, Faculty of Medicine and Health, The University of Sydney, Sydney, NSW Australia; 2 John Walsh Centre for Rehabilitation Research, Northern Sydney Local Health District, St Leonards, Sydney, NSW Australia; 3 The George Institute for Global Health, University of New South Wales, Sydney, Australia; 4 Faculty of Medicine and Health, The University of Sydney, Sydney, NSW Australia; 5 Fiona Stanley Hospital, Murdoch, WA Australia; 6 I.R.C.C.S. Foundation Santa Lucia, Rome, Italy; 7 Department of Life Sciences, Health and Health Professions, Link Camp University, Rome, Italy; 8 Queen Elizabeth National Spinal Injuries Unit, Queen Elizabeth University Hospital, Glasgow, Scotland; 9 The Prince of Wales Hospital, Randwick, NSW Australia; 10 Royal North Shore Hospital, St Leonards, NSW Australia; 11 Royal Rehab, Putney, NSW Australia; 12 Sunnaas Rehabilitation Hospital, Nesodden, Norway; 13 Center of Excellence for Rehabilitation Medicine, University Medical Center Utrecht Brain Center, University Medical Center Utrecht and De Hoogstraat Rehabilitation, Utrecht, the Netherlands; 14 Adelante Centre of Expertise in Rehabilitation and Audiology, Hoensbroek, the Netherlands; and Department of Rehabilitation Medicine, Research School CAPHRI, Maastricht University, Maastricht, the Netherlands; 15 Royal Talbot Rehabilitation Centre, Austin Health, Kew, VIC Australia; 16 The Princess Alexandra Hospital, Brisbane, QLD Australia; 17 London Spinal Cord Injury Centre, Royal National Orthopaedic Hospital Trust, Middlesex, UK; 18 Spinal Cord Injury Rehabilitation, Repat Health Precinct, Daw Park, SA Australia; 19 Ghent University Hospital, Ghent, Belgium; 20 University Hospitals Leuven, Department of Physical and Rehabilitation Medicine, Leuven, Belgium; 21 REVAL, Hasselt University, Diepenbeek, Belgium

**Keywords:** Neurology, Neurological disorders

## Abstract

**Study Design:**

A descriptive quantitative study.

**Objectives:**

To determine how much and what type of exercises and training were provided as part of usual physiotherapy and occupational therapy to participants of the SCI-MT Trial (the Early and Intensive Motor Training for People with Spinal Cord Injuries Trial). This information is important because the SCI-MT Trial concluded that additional motor training is redundant if people with SCI receive equivalent usual care as what was provided to participants of the SCI-MT Trial.

**Settings:**

Fifteen spinal injury units across Europe and Australia.

**Methods:**

Data were collected on the time participants of both groups of the SCI-MT Trial (n = 220) attended physiotherapy and occupational therapy provided as part of usual care over the 10-week intervention period. The International Spinal Cord Injury Physical Therapy and Occupational Therapy Basic Data Set was used to capture time spent on activity and impairment directed categories of exercises and training.

**Results:**

Participants attended a median (interquartile range) of 8.3 (6.2 to 11.4) hours of physiotherapy and occupational therapy per week. Approximately 70% of therapy time was spent actively exercising or training with a median of 3.8 h per week spent on activity-directed and 1.9 h per week spent on impairment-directed exercises and training.

**Conclusions:**

The results of this study detail the amount and type of physiotherapy and occupational therapy that needs to be provided as part of usual rehabilitation care to render additional motor training redundant.

## Introduction

We recently conducted a large international trial designed to determine the effects of 10 weeks of intensive motor training provided soon after spinal cord injury (SCI). It was called The Early and Intensive Motor Training for people with Spinal Cord Injuries Trial (the SCI-MT Trial) and was conducted in 15 sites across Europe and Australia [1]. The primary outcome was total motor scores of the International Standards for the Neurological Classification of SCI and there were a range of other measures of neurological status and function [1]. The results of the trial indicated no benefit on any outcome (except one psychological secondary outcome) from an additional 12 h per week for 10 weeks of motor training targeting muscles at or below the level of injury provided in addition to usual inpatient physiotherapy and occupational therapy [2]. These results challenge the long-held beliefs about activity-dependent spinal plasticity [3]. However, the interpretation of the results rely on a good understanding of the therapy provided to both groups as part of usual inpatient rehabilitation care [4, 5], and specifically the amount and type of exercises and training provided as part of physiotherapy and occupational therapy. For example, the 12 h per week of extra motor training may have been ineffective because of large amounts of therapy both groups received as part of usual care rendering the additional motor training redundant. If this is the case, then the results may not be applicable in situations where patients are receiving less therapy or a different type of therapy than provided to participants in the SCI-MT Trial. The primary purpose therefore of this study was to quantify the amount and type of exercises and training provided as part of usual physiotherapy and occupational therapy to both groups of the SCI-MT Trial to help with the interpretation of the SCI-MT Trial results.

The SCI-MT Trial also provided a unique opportunity to attain data from 15 rehabilitation units across Europe and Australia on the amount and type of exercises and training typically delivered as part of usual inpatient physiotherapy and occupational therapy. These data are important to help others benchmark against because there is surprisingly little comparable data. Furthermore, there are ongoing concerns that people with SCI do not receive enough opportunity for active exercise [6, 7]. Most data on the amount and type of physiotherapy and occupational therapy provided soon after SCI comes from work done by different groups nearly 15 years ago [8–12]. It is not clear how relevant these data are today. Therefore, a secondary purpose of this study was to describe the amount and type of exercises and training provided as part of usual inpatient physiotherapy and occupational therapy across Europe and Australia.

## Methods

Two hundred and twenty people with recent SCI were recruited from 15 sites across Europe and Australia. Participants were eligible for inclusion if they had sustained a SCI within the preceding 10 weeks, were likely to remain in hospital for another 10 weeks and had some motor function below the neurological level (i.e., AIS C or D lesions, and AIS A with motor function more than 3 levels below the motor level) [1]. Written informed consent was obtained from all participants. Participants were randomised to either usual care (Usual Care group) or usual care plus 12 h per week of intensive motor training (Intervention group) [1]. The motor training provided to participants in the Intervention group focused on task-specific training but was supplemented with strength training [13]. It was provided on a one-to-one basis and directed at muscles at, or below, the level of the injury. For the 10-week intervention period, the sites provided comprehensive rehabilitation to all participants of both groups including physiotherapy and occupational therapy. They were instructed not to change their usual rehabilitation practices in response to the trial. The physiotherapy and occupational therapy provided as part of usual rehabilitation included exercises and training as well as other interventions such as stretch, passive movements, equipment prescription, pain management, education and planning for home/work modifications.

Data were collected to capture the following aspects of usual care: the number and hours of therapy sessions scheduled and attended, the number of missed sessions and reasons for missed sessions, and the time in each therapy sessions spent on activity-directed and impairment-directed exercises and training. The next section details how these data were collected.

The start and finish times of all physiotherapy and occupational therapy sessions that were scheduled and delivered over the 10-week intervention period were recorded, irrespective of whether exercises and training were provided. In addition, the following details were recorded: the discipline of the therapist that provided the session (physiotherapy, occupational therapy or other therapist) and the reasons for any missed sessions. Therapists were also asked to record in 5-minute blocks any time devoted to exercises and training that fell into one of the 7 categories of the International Spinal Cord Injury Physical Therapy-Occupational Therapy Basic Data Set - v.1.2 (ISCI PT-OT BDS) [14]. Five of the categories of the ISCI PT-OT BDS capture activity-directed exercises and training which include (i) bed/seated control activities, (ii) standing control activities, (iii) walking, moving up/down stairs, (iv) gross motor upper extremity activities and (v) fine motor upper extremity activities. The other two categories of the ISCI PT-OT BDS capture impairment-directed exercises and training which include (i) strength training and (ii) endurance training (see Supplementary file: Table 1). Therapists were encouraged to record these data directly onto the ISCI PT-OT BDS forms during each therapy session. However, if they preferred, they could keep brief notes and enter the data onto the forms after the session to avoid disrupting therapy. All data were manually recorded in written format by the treating therapist during each session. The exception was one site in which the details of all occupational therapy sessions were self-reported by participants to a research staff member within a day or two of receiving the therapy. Therapists and site principal investigators involved in the trial were trained in all aspects of the trial including the use of ISCI PT-OT BDS. All data were double data entered onto a REDCap Database with resolution of all discrepancies and checks for out-of-range data.

Quantitative data are presented descriptively using means (SD), medians (interquartile ranges), percentages and counts as appropriate. All statistical analyses were performed using Stata Statistical Software V16 [15]. Data are presented for the two groups of the trial as well as for both groups combined.

## Results

A total of 220 participants were recruited and randomised to the SCI-MT Trial. 77% were male and the median (interquartile, IQR) age was 57 years (43 to 67). 60% of participants had American Spinal Injury Association Impairment Scale (AIS) D and 39% of participants had paraplegia (see Table 1 for details). The median (IQR) intervention period over which usual care data were collected was 10.0 weeks (9.3 to 10.3). Four participants were withdrawn from the trial (2 participants from the Intervention group died and 2 participants from the Usual Care group withdrew without explanation). Their data were excluded from this study. There was little differences in the amount and type of therapy provided as part of usual care to the Usual Care group and the Intervention group for any measures of the amount and type of therapy provided as part of usual care so the data of the two groups are presented together unless otherwise stated (See Table 3 for details).

**Table 1:** The characteristics of participants.

	Usual Care group (n = 111)	Intervention group (n = 109)	All participants (n = 220)
**Age, median (IQR)**	54 (42 to 64)	59 (43 to 68)	57 (43 to 67)
**Gender**			
Female, n (%)	23 (21%)	28 (26%)	51 (23%)
Male, n (%)	88 (79%)	81 (74%)	169 (77%)
**Recruitment site**			
Australia, Princess Alexandra Hospital, n (%)	5 (5%)	6 (6%)	11 (5%)
Australia, Royal North Shore Hospital, n (%)	8 (11%)	8 (7%)	16 (7%)
Australia, Royal Rehab, n (%)	1 (1%)	2 (2%)	3 (1%)
Australia, Prince of Wales Hospital, n (%)	8 (7%)	9 (8%)	17 (8%)
Australia, Royal Talbot Rehabilitation Centre, n (%)	7 (6%)	7 (6%)	14 (6%)
Australia, Repat Health Precinct, n (%)	5 (5%)	4 (4%)	9 (4%)
Australia, Fiona Stanley Hospital, n (%)	16 (14%)	16 (15%)	32 (15%)
Scotland, Queen Elizabeth National Spinal Injuries Unit, n (%)	7 (6%)	10 (9%)	17 (8%)
Italy, Foundation Santa Lucia, n (%)	14 (13%)	17 (16%)	31 (14%)
Norway, Sunnaas Rehabilitation Hospital, n (%)	9 (8%)	6 (6%)	15 (7%)
England, Royal National Orthopaedic Hospital NHS Trust, n (%)	7 (6%)	4 (4%)	11 (5%)
Belgium, University Hospital Leuven, n (%)	4 (4%)	2 (2%)	6 (3%)
Belgium, Ghent University Hospital, n (%)	3 (3%)	5 (5%)	8 (4%)
Netherlands, Adelante Hospital, n (%)	8 (7%)	7 (6%)	15 (7%)
Netherlands, Dehoogstraat, n (%)	9 (8%)	6 (6%)	15 (7%)
**Level of injury**			
Tetraplegia, n (%)	66 (60%)	68 (62%)	134 (61%)
Paraplegia, n (%)	45 (41%)	41 (38%)	86 (39%)
**Type of injury (AIS)**^**a**^			
AIS A, n (%)	8 (7%)	4 (4%)	12 (5%)
AIS B, n (%)	1^b^ (1%)	0 (0%)	1 (1%)
AIS C, n (%)	35 (32%)	40 (37%)	75 (34%)
AIS D, n (%)	67 (60%)	65 (60%)	132 (60%)
**Time since injury (months), median (IQR)**	1.5 (1.0 to 1.9)	1.4 (1.2 to 1.8)	1.5 (1.1 to 1.9)
**Time since first sat out of bed (days), median (IQR)**	31 (18 to 48)	32 (21 to 43)	31.5 (20 to 45)

Data are presented by groups and combined.

^a^ASIA impairment scale (AIS).

^b^One person was incorrectly classified as an AIS A. This was identified post randomisation and a decision was made to include the person prior to unblinding.

### Number of therapy sessions

A median (IQR) of 85.0 (63.0 to 111.5) physiotherapy and occupational therapy sessions were recorded (See Table 2 for details). Physiotherapists provided 65% of all therapy sessions (See Table 2 for details). Participants were scheduled to receive a median (IQR) of 9.8 sessions per week (7.2 to 12.5) but attended a median (IQR) of 8.6 sessions per week (6.4 to 11.4) (See Table 2 for details). Therapy sessions were most commonly missed because of appointments (16%), illness (11%) or bladder/bowel problems (9%) (See Table 2 for details). There was a suggestion that the amount of therapy provided over the 10-week period decreased with time, and that people with tetraplegia were scheduled and received more therapy than those with paraplegia (See Supplementary File).

**Table 2:** The details of therapy scheduled and attended by participants and reasons for missed sessions.

	Usual care group (n = 109)		Intervention group (n = 107)		All participants (n = 216)	
	Scheduled	Attended	Scheduled	Attended	Scheduled	Attended
Total number of sessions over intervention period	98 (68 to 126)	87 (64 to 115)	92 (72 to 118)	83 (63 to 107)	92.5 (69 to 122)	85 (63 to 111.5)
Total number of sessions per week	10.4 (7.3 to 12.8)	9.4 (6.6 to 11.8)	9.5 (7.2 to 11.6)	8.5 (6.2 to 10.5)	9.8 (7.2 to 12.5)	8.6 (6.4 to 11.4)
Time (hours) spent in therapy sessions over the intervention period	91.8 (71 to 128)	83 (64.6 to 116)	90.2 (65.2 to 114.8)	76.1 (58.4 to 98)	91.5 (67.2 to 121.8)	80.7 (60.0 to 107.7)
Time (hours) spent in therapy sessions per week	9.5 (7.2 to 14)	8.4 (6.5 to 12.5)	9.3 (6.2 to 12)	7.8 (5.8 to 10.8)	9.3 (6.7 to 12.7)	8.3 (6.2 to 11.4)
**Proportion (%) of therapy sessions provided by:**^a^						
Physiotherapists	62% (51 to 78)	65% (52 to 78)	65% (51 to 79)	65% (52 to 80)	64% (51 to 79)	65% (52 to 80)
Occupational therapists	28% (16 to 37)	28% (16 to 36)	28% (15 to 44)	28% (16 to 44)	28% (16 to 40)	28% (16 to 39)
Other^b^	0% (0 to 14)	0% (0 to 11)	0% (0 to 8)	0% (0 to 8)	0% (0 to 10)	0% (0 to 9)
No discipline specified	0% (0 to 0)	0% (0 to 0)	0% (0 to 0)	0% (0 to 0)	0% (0 to 0)	0% (0 to 0)
Total number of therapy sessions missed over the intervention period	6 (1 to 16)	NA	8 (2 to 13)	NA	7 (2 to 13.5)	NA
No. of therapy sessions missed per week	0.6 (0.1 to 1.5)	NA	0.8 (0.2 to 1.4)	NA	0.7 (0.2 to 1.4)	NA
Proportion (%) of therapy sessions missed per week	6% (2 to 12)	NA	8% (3 to 13)	NA	7% (2 to 13)	NA
**Causes of missed therapy sessions (expressed as proportion of all causes):**						
Appointments	NA	16%	NA	15%	NA	16%
Unwell	NA	9%	NA	13%	NA	11%
Bladder/bowel	NA	8%	NA	9%	NA	9%
Medical tests	NA	7%	NA	7%	NA	7%
Staffing issues	NA	6%	NA	6%	NA	6%
Pain	NA	5%	NA	6%	NA	5%
Fatigue	NA	5%	NA	6%	NA	5%
Nursing issues	NA	4%	NA	4%	NA	4%
Public holiday^c^	NA	4%	NA	3%	NA	4%
Miscellaneous	NA	5%	NA	4%	NA	4%
UTI	NA	3%	NA	4%	NA	3%
Hospital admissions	NA	3%	NA	4%	NA	3%
Outings/ on leave pass	NA	3%	NA	3%	NA	3%
COVID related	NA	2%	NA	3%	NA	3%
Social commitment/ visitors	NA	3%	NA	4%	NA	3%
Declined	NA	2%	NA	2%	NA	2%
Mental health issues	NA	3%	NA	1%	NA	2%
Skin issues	NA	1%	NA	1%	NA	1%
Awaiting on wards for nursing/medical consults or procedures	NA	1%	NA	0%	NA	1%
Asleep	NA	1%	NA	1%	NA	1%
Serious/ adverse events	NA	1%	NA	0%	NA	0%
Not specified	NA	10%	NA	4%	NA	7%

Data are presented by groups and combined. Data are presented as medians (IQR) unless otherwise stated.

^a^Median proportions are calculated separately for each activity and impairment. They therefore do not sum to 100% (unlike mean proportions which are additive).

^b^Other therapists primarily refer to exercise physiologists and allied health assistants.

^c^This reason was probably underestimated as not all sites scheduled therapy sessions on public holidays.

### Time devoted to exercises and training

Participants spent a median (IQR) of 5.7 h (4.3 to 7.8) per week performing exercises or training as captured on the ISCI PT-OT BDS. A median (IQR) of 3.8 h (2.6 to 5.3) per week was spent on activity-directed exercises and training, and 1.9 h (0.9 to 2.9) per week on impairment-directed exercises and training (See Table 3 for details). This equated to a median (IQR) of 36.8 h (25.5 to 51.3) and 18.7 h (8.7 to 29.5) in total over the intervention period, respectively (see Table 3 for details).

**Table 3:** The details of the amount and type of exercises and training provided during all physiotherapy and occupational therapy sessions as captured with the ISCI PT-OT BDS.

	Usual care group (n = 109)		Intervention group (n = 107)		All participants (n = 216)	
Activity-directed exercises	Per week	Over the intervention period	Per week	Over the intervention period	Per week	Over the intervention period
Bed/ seated control activities	0.4 (0.0 to 0.9) 0.9 (1.5)	4.5 (1.3 to 11.2) 10.1 (14.3)	0.3 (0.0 to 1.2) 0.9 (1.2)	4.7 (1.8 to 12.3) 9.4 (11.3)	0.3 (0.0 to 1.0) 0.9 (1.3)	4.6 (1.8 to 11.3) 9.8 (12.9)
Standing control activities	0.9 (0.5 to 1.5) 1.0 (0.8)	8.9 (5.0 to 15.5) 10.5 (7.3)	0.7 (0.3 to 1.3) 0.8 (0.7)	7.3 (3.2 to 12.3) 8.5 (6.9)	0.8 (0.3 to 1.4) 0.9 (0.8)	7.8 (4.2 to 13.6) 9.5 (7.2)
Walking, stairs	0.7 (0.0 to 1.4) 0.8 (0.9)	7.3 (2.1 to 13.2) 8.6 (7.4)	0.5 (0.0 to 0.9) 0.6 (0.6)	5.2 (1.3 to 10.1) 6.1 (5.5)	0.5 (0.0 to 1.2) 0.7 (0.7)	6.4 (1.5 to 11.4) 7.4 (6.7)
Gross motor upper extremity	0.3 (0.0 to 0.7) 0.5 (0.6)	3.7 (1.1 to 7.1) 5.5 (6.1)	0.3 (0.0 to 0.8) 0.4 (0.5)	4.1 (1.3 to 8.0) 5.3 (4.8)	0.3 (0.0 to 0.8) 0.5 (0.6)	3.9 (1.3 to 7.6) 5.4 (5.5)
Fine motor upper extremity	0.0 (0.0 to 1.2) 0.6 (0.9)	2.2 (0.3 to 11.6) 6.1 (7.8)	0.0 (0.0 to 1.0) 0.6 (0.8)	2.3 (0.2 to 9.9) 6.1 (7.4)	0.0 (0.0 to 1.1) 0.6 (0.8)	2.2 (0.3 to 10.9) 6.1 (7.6)
*Total time (hours)*^a^	*4.2 (2.8 to 5.7)* *4.2 (2.1)*	*38.8 (25.8 to 56.7)* *40.8 (19)*	*3.6 (2.5 to 4.9)* *3.7 (1.6)*	*33.8 (24.7 to 46.2)* *35.4 (14.5)*	*3.8 (2.6 to 5.3)* *3.9 (1.8)*	*36.8 (25.5 to 51.3)* *38.1 (17.1)*
**Impairment-directed exercises**						
Strength training	0.4 (0.0 to 0.9) 0.9 (1.5)	14.3 (7.8 to 24.5) 16.7 (12.4)	0.3 (0.0 to 1.2) 0.9 (1.2)	13.8 (6.3 to 21.8) 15.0 (11.9)	0.3 (0.0 to 1.0) 0.9 (1.3)	13.9 (7.0 to 22.8) 15.9 (12.1)
Endurance training	0.9 (0.5 to 1.5) 1.0 (0.8)	4.0 (0.8 to 8.9) 5.7 (5.8)	0.7 (0.3 to 1.3) 0.8 (0.7)	2.6 (0.8 to 7.7) 5.1 (6.3)	0.8 (0.3 to 1.4) 0.9 (0.8)	3.4 (0.8 to 8.6) 5.4 (6.0)
*Total time (hours)*^a^	*2.0 (0.9 to 3.3)* *2.3 (1.8)*	*19.2 (9.4 to 32.8)* *22.4 (16.5)*	*1.8 (0.8 to 2.8)* *2.0 (1.7)*	*18.2 (7.9 to 26.8)* *20.2 (16.4)*	*1.9 (0.9 to 2.9)* *2.2 (1.8)*	*18.7 (8.7 to 29.5)* *21.3 (16.4)*
**Activity-directed and Impairment-directed exercises**						
*Total time (hours)*^a^	*6.2 (4.5 to 8.3)* *6.7 (3.2)*	*61.1(44.2 to 80.0)* *63.2 (29.6)*	*5.3 (3.9 to 7.2)* *5.8 (2.7)*	*52.2 (38.9 to 68.3)* *55.6 (25.2)*	*5.7(4.3 to 7.8)* *6.2 (3.0)*	*55.5 (39.9 to 75.1)* *59.4 (27.7)*

Data are presented by groups and combined (n = 220). Data are presented as median (IQR) and mean (SD).

^a^Medians are not additive; therefore the median totals do not equal the sum of the component medians.

## Discussion

This study describes the amount and type of exercises and training that were provided as part of usual physiotherapy and occupational therapy to participants of the SCI-MT Trial. This information is important because the SCI-MT Trial found no effect from the intensive motor training provided in addition to usual inpatient rehabilitation care. However, these results cannot be generalised to situations in which the amount and type of physiotherapy and occupational therapy provided to patients is different to that provided in the SCI-MT Trial [4, 11, 16]. For example, additional motor training may be effective in situations where the physiotherapy and occupational therapy is less or different to that provided in the SCI-MT Trial. It is important to understand the usual therapy provided to both groups in the SCI-MT Trial to explain the results of the trial. For instance, our failure to demonstrate a treatment effect could be explained by therapists inadvertently providing more therapy or a different type of therapy to participants in the Usual Care group than participants in the Intervention group in response to the trial. However, there was no indication that this was the case. The amount and type of exercises and training provided in physiotherapy and occupational therapy as part of usual care were similar for both groups, and participants of both groups spent comparable amounts of time attending therapy.

The results of this study not only help with the interpretation of the SCI-MT Trial but they also provide rare recent data on the amount of physiotherapy and occupational therapy provided to people with SCI. For example, our results indicate that participants spent a median of 8.3 h per week in physiotherapy and occupational therapy. These results suggest an increase in the amount of therapy to what was reported in 2011 at which time people from SCI units in the Netherlands, Norway and Australia were reportedly receiving approximately 4 to 6 h per week [10, 11]. However, another study also done in 2011 suggested that people with SCI in the USA were receiving substantially more time in physiotherapy and occupational therapy. Namely 14.1 h per week of physiotherapy and occupational therapy [8, 9] (compared to our 8.3 h). We don’t know if and how the amount of therapy provided in 2011 in the USA has changed over the last 15 years although lengths of hospital stay in the USA are generally shorter than lengths of hospital stay in Australia and European countries [17–19]. So possibly the total time in physiotherapy and occupational therapy over the course of a patient’s hospital stay is similar. And of course it is very difficult to make comparisons because different health systems have different financial reimbursements for therapy. In addition, different studies collect and record data in different ways and in people at different stages of rehabilitation.

It is surprising that 25% of participants attended physiotherapy and occupational therapy for less than 6.2 h per week and only spent 4.3 h per week on any exercises or training captured on the ISCI PT-OT BDS (see Tables 2 and 3 for details). This is a small amount of therapy particularly considering that these were people with recent SCI undergoing specialist rehabilitation. While these figures justify the recent concern that people with SCI are not receiving sufficient therapy [17], it could also be argued that these concerns are not justified because the results of the SCI-MT Trial indicate no benefit from additional therapy time. However, it is important to recognise that the additional therapy provided as part of the SCI-MT Trial was only motor training directed at, or below the level of the injury [1, 13]. The results of the SCI-MT Trial do not tell us about the possible benefits of additional time spent in physiotherapy and occupational therapy that is not specifically focused on the type of motor training provided in the SCI-MT Trial. For example, patients may benefit from more than 6.2 h per week of physiotherapy and occupational therapy that focuses on other aspects of rehabilitation including learning independence and skills associated with seated and/or upright mobility. There is clearly a need for clarity around optimal amounts of therapy for people with recent SCI, and this needs to be underpinned by high quality clinical trials or sophisticated analyses of cohort data. In the meantime, it may also be worthwhile to explore ways to minimise the number of missed therapy sessions. Some missed sessions are clearly unavoidable (e.g. 11% of missed sessions were due to participants being unwell). However, 16% of missed sessions were because of conflicting appointments. This may also be unavoidable but it would be worth investigating further to see if anything could be done to minimise these.

It is often assumed that there is a lot of wasted time in therapy [6, 7]. However, to the contrary, our data indicates that participants spent 70% of their time in physiotherapy or occupational therapy engaged in exercises that were captured on the ISCI PT-OT BDS. This was equivalent to 5.7 h per week (IQR, 4.3 to 7.8). This leaves 30% of time in physiotherapy and occupational therapy on all the many other aspects of therapy including assessments, equipment prescription, patient education, revision of home and work environments, treatments for pain and range of motion, and time spent on goal setting. It therefore appears that therapists and people with SCI are using their time in therapy productively with very little “down time”.

Participants devoted more time per week on exercises and training classified as activity-directed than impairment-directed (3.8 h versus 1.9 h), even though more time (0.3 h per week) was spent on strength training than any other type of intervention captured on the ISCI PT-OT BDS. This emphasis on activity-directed rather than impairment-directed interventions reflects the current focus on the practice of functional activities [20–22]. This seems reasonable and justified. However, it does raise questions about the amount of strength training provided each week (0.3 h per week; i.e., 18 min) and how this could be sufficient and effective particularly when people often have weakness in a lot of different muscles, and current guidelines indicate that strength training needs to be conducted at least two times, and preferably three times per week [23, 24]. Possibly, these figures underestimate the amount of strength training that was provided because of misclassification. The distinction between strength training and other types of exercises and training can be ambiguous and is not fully addressed in the definitions provided within the ISCI PT-OT BDS. Nonetheless, there could be little doubt that strength is a key impairment limiting people’s abilities to move. So whilst the practice of functional activities may have carryover effects on strength, it would seem prudent to also be directing concentrated effort on strength training (particularly for those with grade 3 or greater strength in view of the evidence about the effectiveness of progressive resistance training in these muscles) [23, 25].

Whilst the results of this study could (and should) be used by SCI units around the world to reflect on and benchmark the therapy they provide, care needs to be taken before generalising the results of this study to all SCI units around the world. The results may not reflect usual physiotherapy and occupational therapy everywhere. So even though we included 15 different sites from 6 countries across Europe and Australia, these SCI units may not be representative of all SCI units around the world. The SCI units included in the SCI-MT Trial were selected because they had similar approaches to rehabilitation (and similar lengths of hospital stay). This was done in an attempt to minimise differences between sites; an important consideration for a trial in which all participants received usual care. However, if we had set out to determine usual care around the world then it would have been better if we either randomly selected SCI units from around the world or selected sites with different approaches to rehabilitation. This would have provided a more representative sample. Importantly, we did not set out to compare the type or amount of usual care provided by the 15 sites. We only set out to quantify the usual care provided in the SCI-MT Trial so others could compare their own usual care with that provided in our trial to make a decision about whether the results of the SCI-MT Trial apply to them.

We did not distinguish between exercises and training directed at, above or below the level of injury even though this information may have been helpful. Similarly, we did not collect data on all the different interventions provided as part of physiotherapy and occupational therapy that were not captured on the ISCI PT-OT BDS. We were aware of these limitations prior to the commencement of the SCI-MT Trial but we needed to weigh up the possible benefits of these data with the associated time and cost of collecting them. Ultimately, we decided that these costs were too high and we were more likely to get better and more accurate data if we minimised the burden of data collection on therapists. Even still, the data collection was an additional task for busy therapists and consequently they may not have always accurately recorded the therapy they provided despite their best attempts. In addition, some sites also provided group exercise sessions that were not always recorded. So, our data may underestimate the therapy that was provided but nonetheless provides the most comprehensive recent data on current practices.

In all, the results of this study are not only important for the interpretation of the SCI-MT Trial but they also provide useful data about the amount and type of physiotherapy and occupational therapy typically provided by SCI units across Europe and Australia. We hope that these data will prompt SCI units and researchers to reflect on the optimal amount and combination of different types of therapies provided to people with recent SCI. More work in this area will lead to a better understanding of what should be provided to lead to better outcomes for people with SCI.

## Supplementary information

Supplementary File

## Data Availability

Data are available upon reasonable request. Deidentified participant data may be accessed by researchers who provide a methodological proposal directed to the corresponding author, LAH. Approval for data access will be granted on a case-by-case basis at the discretion of the principal investigator, LAH. The data will be accessible from the date of this article’s publication and will be available for a period of 5 years thereafter.
