# Supplementary material for: How much and what type of exercises and training were provided to people with spinal cord injury as part of usual physiotherapy and occupational therapy in the SCI-MT Trial?
Source: Spinal Cord. 2026 Feb 27;64(4):317–23. doi: 10.1038/s41393-026-01180-7 (PMC13065470; doi:10.1038/s41393-026-01180-7)
Supplement: Supplementary file 1 — Supplementary File [file 41393_2026_1180_MOESM1_ESM.pdf]

Supplementary File Table 1: Form used by therapists to capture time devoted to exercises and training that fell into one of the 7 categories of the International Spinal Cord Injury Physical Therapy-Occupational Therapy Basic Data Set (ISCI PT-OT BDS) [14].

**PARTICIPANT INITIALS:**						**PARTICIPANT ID:**			-				
							(site number)			(participant ID)			
**WEEK STARTING:**	d	d	m	m	y	y	**SESSION:**	Usual care		Additional research therapy			(circle one)

M T W Th F Sat Sun	PT OT Other	Session Start time:	Session Finish time:	Missed all	Missed >15 min
(a) Bed/seated control activities: balance, seated transfers, bed mobility					Record time to nearest 5 minutes
(b) Standing control activities: standing, balance, standing transfers weight bearing					
(c) Walking, stairs (inside, outside)					
(d) Gross motor upper extremity: dressing, washing, manual wheelchair mobility					
(e) Fine motor upper extremity: grooming, self-feeding, buttoning, zipping, adjustment of clothing					
(f) Strength training (including electrical stimulation for strength)					
(g) Endurance training (including electrical stimulation for endurance)					

M T W Th F Sat Sun	PT OT Other	Session Start time:	Session Finish time:	Missed all	Missed >15 min
(a) Bed/seated control activities: balance, seated transfers, bed mobility					Record time to nearest 5 minutes
(b) Standing control activities: standing, balance, standing transfers weight bearing					
(c) Walking, stairs (inside, outside)					
(d) Gross motor upper extremity: dressing, washing, manual wheelchair mobility					
(e) Fine motor upper extremity: grooming, self-feeding, buttoning, zipping, adjustment of clothing					
(f) Strength training (including electrical stimulation for strength)					
(g) Endurance training (including electrical stimulation for endurance)					

M T W Th F Sat Sun	PT OT Other	Session Start time:	Session Finish time:	Missed all	Missed >15 min
(a) Bed/seated control activities: balance, seated transfers, bed mobility					Record time to nearest 5 minutes
(b) Standing control activities: standing, balance, standing transfers weight bearing					
(c) Walking, stairs (inside, outside)					
(d) Gross motor upper extremity: dressing, washing, manual wheelchair mobility					
(e) Fine motor upper extremity: grooming, self-feeding, buttoning, zipping, adjustment of clothing					
(f) Strength training (including electrical stimulation for strength)					
(g) Endurance training (including electrical stimulation for endurance)					

M T W Th F Sat Sun	PT OT Other	Session Start time:	Session Finish time:	Missed all	Missed >15 min
(a) Bed/seated control activities: balance, seated transfers, bed mobility					Record time to nearest 5 minutes
(b) Standing control activities: standing, balance, standing transfers weight bearing					
(c) Walking, stairs (inside, outside)					
(d) Gross motor upper extremity: dressing, washing, manual wheelchair mobility					
(e) Fine motor upper extremity: grooming, self-feeding, buttoning, zipping, adjustment of clothing					
(f) Strength training (including electrical stimulation for strength)					
(g) Endurance training (including electrical stimulation for endurance)					

M T W Th F Sat Sun	PT OT Other	Session Start time:	Session Finish time:	Missed all	Missed >15 min
(a) Bed/seated control activities: balance, seated transfers, bed mobility					Record time to nearest 5 minutes
(b) Standing control activities: standing, balance, standing transfers weight bearing					
(c) Walking, stairs (inside, outside)					
(d) Gross motor upper extremity: dressing, washing, manual wheelchair mobility					
(e) Fine motor upper extremity: grooming, self-feeding, buttoning, zipping, adjustment of clothing					
(f) Strength training (including electrical stimulation for strength)					
(g) Endurance training (including electrical stimulation for endurance)					

M T W Th F Sat Sun	PT OT Other	Session Start time:	Session Finish time:	Missed all	Missed >15 min
(a) Bed/seated control activities: balance, seated transfers, bed mobility					Record time to nearest 5 minutes
(b) Standing control activities: standing, balance, standing transfers weight bearing					
(c) Walking, stairs (inside, outside)					
(d) Gross motor upper extremity: dressing, washing, manual wheelchair mobility					
(e) Fine motor upper extremity: grooming, self-feeding, buttoning, zipping, adjustment of clothing					
(f) Strength training (including electrical stimulation for strength)					
(g) Endurance training (including electrical stimulation for endurance)					

Figure 1: The time (hours) per week over the 10-week trial period that participants (i) were scheduled to attend therapy, (ii) attended therapy, and (iii) were actively doing exercises.

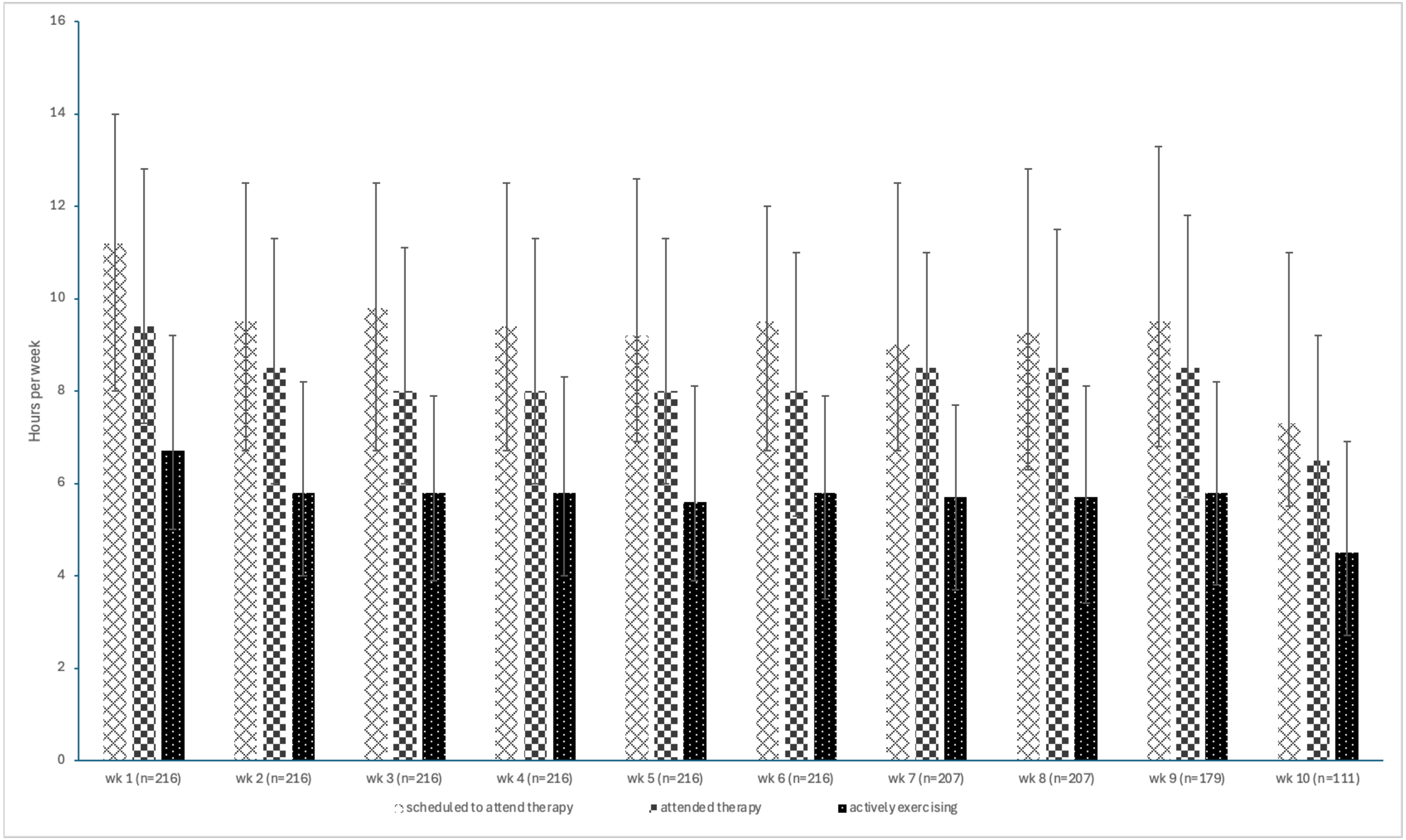

Table 2: The details of the amount of exercises and training provided during all physiotherapy and occupational therapy sessions as captured with the ISCI PT-OT BDS during the 10-week trial period. Data are presented as median (IQR) and separately for people with tetraplegia and paraplegia.

	Time scheduled to attend therapy		Time attended therapy		Time actively exercising	
**Level of injury**	**Tetraplegia**	**Paraplegia**	**Tetraplegia**	**Paraplegia**	**Tetraplegia**	**Paraplegia**
Week 1	11.9 (8.7 to 14.8) (n = 130)	9.6 (7.7 to 13.3) (n = 86)	10.1 (7.5 to 13.8) (n = 130)	8.5 (7.0 to 11.5) (n = 86)	7.0 (5.3 to 9.7) (n = 130)	6.2 (4.5 to 8.1) (n = 86)
Week 2	10.5 (8.0 to 13.0) (n = 130)	8.2 (6.0 to 11.5) (n = 86)	9.5 (6.8 to 11.5) (n = 130)	7.0 (5.5 to 10.2) (n = 86)	6.6 (4.4 to 8.7) (n = 130)	5.0 (3.4 to 7.2) (n = 86)
Week 3	11.2 (7.6 to 12.5) (n = 130)	7.7 (5.5 to 10.5) (n = 86)	9.5 (6.7 to 12.0) (n = 130)	7.0 (5.0 to 9.0) (n = 86)	6.4 (4.4 to 8.4) (n = 130)	5.0 (3.2 to 6.3) (n = 86)
Week 4	11.0 (7.5 to 13.5) (n = 130)	7.9 (5.8 to 10.8) (n = 86)	9.0 (7.0 to 12.5) (n = 130)	7.0 (5.0 to 9.8) (n = 86)	6.6 (4.7 to 8.6) (n = 130)	5.0 (3.4 to 6.5) (n = 86)
Week 5	10.5 (7.7 to 14.0) (n = 130)	8.0 (5.3 to 10.0) (n = 86)	8.5 (6.7 to 12.2) (n = 130)	6.7 (5.0 to 9.8) (n = 86)	6.2 (4.5 to 8.8) (n = 130)	4.9 (3.2 to 7.0) (n = 86)
Week 6	10.1 (7.7 to 13.2) (n = 130)	8.0 (5.7 to 10.5) (n = 86)	9.1 (6.0 to 12.0) (n = 130)	6.7 (4.0 to 10.0) (n = 86)	6.2 (4.1 to 8.4) (n = 130)	4.2 (2.8 to 7.2) (n = 86)
Week 7	10.5 (8.0 to 13.8) (n = 130)	7.2 (5.0 to 10.2) (n = 86)	9.0 (6.8 to 12.5) (n = 130)	6.0 (4.1 to 9.2) (n = 86)	6.2 (4.5 to 8.4) (n = 130)	4.5 (2.4 to 6.2) (n = 86)
Week 8	10.5 (7.6 to 14.0) (n = 126)	7.5 (5.0 to 10.0) (n = 81)	9.1 (7.0 to 12.0) (n = 126)	6.6 (4.6 to 9.5) (n = 81)	6.2 (4.2 to 9.2) (n = 126)	5.1 (2.2 to 7.1) (n = 81)
Week 9	10.0 (7.8 to 14.0) (n = 112)	8.2 (5.0 to 10.5) (n = 67)	9.0 (6.6 to 12.9) (n = 112)	7.5 (4.8 to 10.0) (n = 67)	6.2 (4.6 to 8.8) (n = 112)	4.7 (2.9 to 6.6) (n = 67)
Week 10	8.5 (6.5 to 12.4) (n = 69)	6.0 (4.4 to 8.0) (n = 42)	7.8 (5.3 to 11.5) (n = 69)	5.6 (3.0 to 7.0) (n = 42)	5.2 (3.0 to 7.6) (n = 69)	3.5 (2.2 to 5.3) (n = 42)

Table 3: The details of the amount and type of exercises and training provided during all physiotherapy and occupational therapy sessions as captured with the ISCI PT-OT BDS during the 10-week trial period. Data are presented as median (IQR) and mean (SD), and separately for people with tetraplegia and paraplegia.

	**Tetraplegia** (n = 130)	**Paraplegia** (n = 86)
**Activity-directed exercises**		
Bed/ seated control activities	3.9 (1.6 to 9.3)<br>9.0 (13.1)	6.1 (2.6 to 15.3)<br>10.9 (12.6)
Standing control activities	8.4 (5.0 to 13.8)<br>9.8 (6.9)	6.9 (3.2 to 13.0)<br>9.0 (7.6)
Walking, stairs	6.5 (1.9 to 10.9)<br>7.1 (6.2)	6.3 (1.2 to 13.4)<br>7.7 (7.3)
Gross motor upper extremity	6.0 (2.8 to 9.3)<br>6.9 (5.9)	1.8 (0.3 to 4.6)<br>3.1 (3.7)
Fine motor upper extremity	9.5 (3.5 to 14.6)<br>9.9 (7.7)	0.0 (0.0 to 0.5)<br>0.4 (0.8)
Total time (hours) ‡	*41.8 (30.4 to 53.8)*<br>*42.8 (16.7)*	*28.6 (18.3 to 42.2)*<br>*31.1 (15.4)*
**Impairment-directed exercises**		
Strength training	15.1 (8.5 to 24.5)<br>17.4 (12.4)	11.3 (5.0 to 19.8)<br>13.6 (11.5)
Endurance training	3.7 (1.1 to 8.8)<br>6.0 (6.6)	2.7 (0.5 to 7.2)<br>4.5 (4.9)
Total time (hours) ‡	*20.1 (13.4 to 32.2)*<br>*23.4 (17.0)*	*15.8 (7.3 to 26.0)*<br>*18.1 (15.0)*

‡ Medians are not additive; therefore the median totals do not equal the sum of the component medians).
